# Supplementary material for: How does the development of the digital economy in RCEP member countries affect China’s cross-border e-commerce exports?
Source: PLoS One. 2024 Dec 23;19(12):e0310975. doi: 10.1371/journal.pone.0310975 (PMC11666022; doi:10.1371/journal.pone.0310975)
Supplement: Attachment — Submitted filename: PONE-D-24-19141_Review.pdf [file pone.0310975.s001.pdf]

Referee Report on:

How does the development of the digital economy in RCEP member countries affect China's cross-border e-commerce exports?

This paper takes up an interesting issue, relating China's cross-border e-commerce export performances to digitalization of rest of REEP member countries by utilizing multi-dimensional panel data from 2012 to 2021. The research results show that, in terms of direct impact, the development of the digital economy in RCEP member countries has promoted China's cross-border e-commerce export, and its impact is heterogeneous. Regarding indirect impact, improving the quality of RCEP member countries' transportation infrastructure and institutional quality are moderating factors promoting China's e-commerce export.

Comments:

1. Table 1 has constructed index weights for evaluation indicators for the digital economy development level of RCEP member countries using Entropy Method. But the readers can't get any insight from table 1 how is this done! Even a small note on the mechanism in the form of an appendix could have been better.
2. The paper uses "Economic Freedom" as an indicator of institutional quality! But World Bank Group's databank provides a separate "institutional quality" indicator under the head of "Worldwide Governance Indicators". If data for RECP member countries are available for the study period, the authors should use that(those) indicator(s), instead of "Economic Freedom" which is more general in nature.
3. In table 3, should it be "sample size" or number of observations! The paper uses macro and institutional data and hence nothing is taken as "sample".
4. In table 4, the results are very good. "Dei", "Open" and "Dis" are strongly affecting e-com exports but "GDP" and "Tar" are having relatively lesser effects. "Tar" effect is understandable perhaps because tariff rates are pretty low and/or unchanging but why "Pop" is having a weak effect – what could be reasons for that?
5. What is digital economy readiness index (Der)? How is it constructed or what is its data source?
6. In line 560 – 62, it is said: "It uses the fixed telephone subscriptions per 100 inhabitants in RCEP member countries in 2007 as an instrumental variable to measure the level of digital economic development among RCEP member countries." I wonder whether this is a proper proxy measurement of level of digital economic development among RCEP member countries for the period 2012 – 21.
7. In conclusion at line 767, it is said that "……optimize the domestic digital economy environment." Assuming it is suggested for China, did the author test how can China's enhanced digitalization positively affect its own e-com export? Or is it a mere suggestion. I think the conclusion can be properly presented based on the findings of the empirical exercises.
8. Did the author provide data source for "the quality of transportation infrastructure" and "economic freedom"? I might have missed as couldn't find!

9. Finally and most importantly, the data period is very interesting! Given that the time period is 2012 – 21 and COVID took place in between, if further data are available till 2023 – a very interesting exercise could have been the pre and post COVID differences with an effort to identify the presence of structural break. Two reasons for that – (a) China and most of the RCEP members were the worst victims of the viral outbreak and (b) post COVID, people were forced to develop a better digital technology in every field of life including trade practices. So it could have been quite interesting to see how did the interplay affect the outcome as the supply chain must have been affected.
