# Supplementary material for: How does the development of the digital economy in RCEP member countries affect China’s cross-border e-commerce exports?
Source: PLoS One. 2024 Dec 23;19(12):e0310975. doi: 10.1371/journal.pone.0310975 (PMC11666022; doi:10.1371/journal.pone.0310975)
Supplement: Attachment — Submitted filename: Response to Reviewers.docx [file pone.0310975.s002.docx]

**Response to Reviewers**

**1.Table 1 has constructed index weights for evaluation indicators for the digital economy development level of RCEP member countries using Entropy Method. But the readers can’t get any insight from table 1 how is this done! Even a small note on the mechanism in the form of an appendix could have been better.**

In section 4.1, the calculation steps of the entropy method have been added to explain how the index weights and comprehensive scores for the digital economy development level are calculated.

**2.The paper uses “Economic Freedom” as an indicator of institutional quality! But World Bank Group’s databank provides a separate “institutional quality” indicator under the head of “Worldwide Governance Indicators”. If data for RECP member countries are available for the study period, the authors should use that(those) indicator(s), instead of “Economic Freedom” which is more general in nature.**

Based on the reviewers' suggestions, the institutional quality indicators from the World Bank's Worldwide Governance Indicators database have been adopted in section 5.1 to replace the original economic freedom indicators. Corresponding changes have been made to the variables in the model.

**3.In table 3, should it be “sample size” or number of observations! The paper uses macro and institutional data and hence nothing is taken as “sample”.**

Based on the reviewers' comments, the "sample size" in Table 3 of section 5.2 has been revised to "Number of observations".

**4.In table 4, the results are very good. “Dei”, “Open” and “Dis” are strongly affecting e-com exports but “GDP” and “Tar” are having relatively lesser effects. “Tar” effect is understandable perhaps because tariff rates are pretty low and/or unchanging but why “Pop” is having a weak effect – what could be reasons for that?**

Section 5.3.1 provides further explanation and clarification on the potential reasons for the relatively weak impact of the population size factor (Pop) on China's cross-border e-commerce exports.

**5.What is digital economy readiness index (Der)? How is it constructed or what is its data source?**

In section 5.3.2, the Digital Economy Readiness Index has been corrected to the Network Readiness Index. Additionally, an explanation and clarification regarding the data sources and indicator composition of the Network Readiness Index have been provided.

**6.In line 560 – 62, it is said: “It uses the fixed telephone subscriptions per 100 inhabitants in RCEP member countries in 2007 as an instrumental variable to measure the level of digital economic development among RCEP member countries.” I wonder whether this is a proper proxy measurement of level of digital economic development among RCEP member countries for the period 2012 – 21.**

In section 5.3.2, the discussion of endogeneity and the rationale and logic for using the 2007 fixed telephone subscriptions per 100 inhabitants in RCEP member countries as an instrumental variable to measure the level of digital economic development in RCEP member countries have been revised and improved.

**7.In conclusion at line 767, it is said that “……optimize the domestic digital economy environment.” Assuming it is suggested for China, did the author test how can China’s enhanced digitalization positively affect its own e-com export? Or is it a mere suggestion. I think the conclusion can be properly presented based on the findings of the empirical exercises.**

Based on the reviewers' comments and the empirical research findings of the paper, the third part of the conclusions and recommendations has been revised and improved accordingly.

**8.Did the author provide data source for “the quality of transportation infrastructure” and “economic freedom”? I might have missed as couldn’t find!**

The data sources for the transportation infrastructure quality indicators and institutional quality indicators have been added to section 5.1.

**9. Finally and most importantly, the data period is very interesting! Given that the time period is 2012 – 21 and COVID took place in between, if further data are available till 2023 – a very interesting exercise could have been the pre and post COVID differences with an effort to identify the presence of structural break. Two reasons for that – (a) China and most of the RCEP members were the worst victims of the viral outbreak and (b) post COVID, people were forced to develop a better digital technology in every field of life including trade practices. So it could have been quite interesting to see how did the interplay affect the outcome as the supply chain must have been affected.**

In Section 5.5, an analysis of the impact of the COVID-19 pandemic has been added to discuss how the development of the digital economy among RCEP member countries affects the changes in China's cross-border e-commerce exports against the backdrop of the pandemic outbreak. Due to data limitations, the study only extended the research data beyond 2023, focusing primarily on analyzing the impact of changes in China's cross-border e-commerce exports from 2020 to 2021 due to the development of the digital economy among RCEP member countries post the pandemic outbreak.
