# Supplementary material for: How does the development of the digital economy in RCEP member countries affect China’s cross-border e-commerce exports?
Source: PLoS One. 2024 Dec 23;19(12):e0310975. doi: 10.1371/journal.pone.0310975 (PMC11666022; doi:10.1371/journal.pone.0310975)
Supplement: Attachment — Submitted filename: Response to Reviewers.docx [file pone.0310975.s004.docx]

**Reviewer 1**

**1.First of all, the Abstract should not contain any unidentified terms such as RCEP, though it is a popular term.**

The abstract section has been revised.

**2.The theoretical conceptualisation is not sufficient. As the study has contained many explanatory and control variables, I suggest the theoretical underpinning should be such that it can explain the core relationships among the variables capturing all of them. The authors may also use the functional forms of the interrelationships among the variables.**

The research focus of this paper has been clarified before Section 3.1. Additionally, the relationships between the digital economy development of RCEP member countries, China's cross-border e-commerce exports, and the moderating variables (transportation infrastructure quality and institutional quality) have been further elucidated.

**3.The study should categorically explain the motivation and importance of the study. Having a moderate score in e commerce index for China, why the authors have considered China, not even Singapore like countries who have very high index values. It should use updated research articles to find the appropriate research gaps.**

The final paragraph of Section 1, Introduction, has been revised and supplemented to clarify the motivation and significance of this study. Although China's digital economy development score is low, as a core member of RCEP, China has actively participated in formulating and signing the agreement. Compared to countries with higher digital economy development scores, China's cross-border e-commerce exports significantly promote international trade and economic cooperation. Therefore, as clarified in the Introduction, this paper selects China as the research subject. Based on the reviewer's suggestions, the latest research literature has also been incorporated into the literature review section.

**4.As the study largely depends on the index score of the digital economy of the member countries, the authors should discuss the method of index computations in detail to ease the readability of the readers from the multi-disciplinary areas.**

In section 4.1, the calculation steps of the entropy method have been added to explain how the index weights and comprehensive scores for the digital economy development level are calculated.

**5.Before going to have the econometric estimations in the panel data format, the authors could have tried for pair-wise correlation analysis to have a primary view on the degree of associations among the variables.**

Section 5.3.1 has been expanded to examine the correlation and multicollinearity of the empirical data. The results of these tests have been analyzed and explained to elucidate the relationships among the data.

**6.As having variations in the index scores among the group of RCEP, the authors may segregate/cluster the countries in two different panels to carry out the similar econometric exercise to have better results for two sets of countries to have proper policy formulations.**

Section 5.4.3 has been expanded to include an analysis of the heterogeneity in digital economy development levels. Using the previously discussed digital economy development scores, RCEP member countries were categorized into high and low digital economy development groups for empirical testing. The results were compared to explore the differences and analyze the potential reasons for these discrepancies.

**Journal Requirements:**

**Please review your reference list to ensure that it is complete and correct. If you have cited papers that have been retracted, please include the rationale for doing so in the manuscript text, or remove these references and replace them with relevant current references. Any changes to the reference list should be mentioned in the rebuttal letter that accompanies your revised manuscript. If you need to cite a retracted article, indicate the article’s retracted status in the References list and also include a citation and full reference for the retraction notice.**

The references were carefully checked and revised.
